# In situ reconstruction of vascular inflow/outflow to left lateral liver section, ex-vivo liver resection and autologous liver transplantation of remaining liver remnant for hepatic alveolar echinococcosis

**DOI:** 10.1016/j.ijscr.2020.03.023

**Published:** 2020-03-28

**Authors:** Yu Zhang, Eric C.H. Lai, Chong Yang, Hongji Yang, Jun Liu, Guo Zhou, Di Xian, Shaoping Deng, Wan Yee Lau

**Affiliations:** a Organ Transplantation Center, Sichuan Academy of Medical Sciences & Sichuan Provincial People's Hospital, School of Clinical Medicine, University of Electronic Science and Technology of China, Chengdu 610072, Sichuan, China; b Ultrasonography Department, Sichuan Academy of Medical Sciences & Sichuan Provincial People's Hospital, School of Clinical Medicine, University of Electronic Science and Technology of China, Chengdu 610072, Sichuan, China; c Faculty of Medicine, The Chinese University of Hong Kong, Prince of Wales Hospital, Shatin, N.T., Hong Kong SAR, China

**Keywords:** Hepatic alveolar echinococcosis, Auxiliary autologous liver transplantation, Ex-vivo liver resection, Hepatic vein reconstruction

## Abstract

**Objective:**

This is a case report on a patient with advanced hepatic alveolar echinococcosis (HAE) treated with autologous liver transplantation without any veno-venous bypass using the modified technique of ex vivo liver resection and autologous liver transplantation (the ERAT technique).

**Method:**

A 27-year old male with advanced HAE underwent in situ reconstruction of vascular inflow/outflow to left lateral liver section, ex-vivo liver resection and autologous liver transplantation of remaining liver remnant (the modified ERAT technique). The operation consisted of hepatotomy along the right border of the falciform ligament, reconstruction of portal vein supplying the left lateral liver section, reconstruction of left hepatic vein, followed by removal of liver segments S1, S4 to S8, ex vivo resection of all involved tissues within these liver segments in the liver remnant, and autologous liver transplantation of the resected liver remnant. The whole surgical procedure lasted for 12 h, and the blood lost was 800 mL. The patient recovered uneventfully in the post-operation period.

**Conclusion:**

The in situ reconstruction of the vascular inflow/outflow of left lateral liver section maintained the PV circulation and provided liver functional support during the operation. The subsequent autologous liver transplantation provided additional liver functional tissues, thus reduced the risk of post-hepatectomy liver failure. This surgical procedure did not require any veno-venous bypass.

## Highlights

• Advanced HAE treated with the modified technique of ex vivo liver resection and autologous liver transplantation.
• The in situ reconstruction of the vascular inflow/outflow of left lateral liver section maintained the PV circulation.
• The subsequent autologous right liver transplantation provided additional liver functional tissues, thus reduced the risk of liver failure.
• This surgical procedure did not require any veno-venous bypass.

## 1 Introduction

Hepatic alveolar echinococcosis (HAE) is a life-threatening disease characterized with a long incubation period, with invasion of multiple intrahepatic structures, resulting in difficulties in resection [1]. Previously, orthotopic liver transplantation (LT) with allografts have been used as a life-saving procedure for ‘unresectable’ cases. However, donor liver graft shortage, high recurrence rates and the need for long-term immunosuppressive therapy led to its limited utilization for end-stage HAE [[2], [3], [4], [5], [6]]. As HAE is a benign disease, ex-vivo liver resection and autologous liver transplantation (ERAT) have subsequently been reported to treat end-stage HAE [[7], [8], [9], [10], [11]].

Comparing with orthotopic LT using allografts, ERAT avoids immunosuppressive therapy, thus reduces the risk of recurrence and metastasis after operation [7]. Up to now, we have treated 27 patients with HAE using ERAT. In this case report which involved a modified ERAT procedure, we adopted the principle of in vivo liver split [12] and reconstructed the portal vascular inflow and hepatic venous outflow of the left-lateral liver section. The remaining liver was removed using the ex vivo technique and bench surgery was done to resect all the involved parts of the liver by HAE, followed by autologous liver transplantation. The modified ERAT procedure preserved the portal venous circulatory states and part of the liver function during surgery. There was an anhepatic phase for 1 h and veno-venous bypass was not required. This work has been reported in line with the SCARE criteria [13].

## 2 Case report

A 27-old man of Han nationality presented to our center because of multiple hepatic lesions detected on physical examination in January 2019. The patient had lived in an endemic AE area. AE was diagnosed in this patient by a positive indirect echinococcal hemagglutination test, a positive anti-E granulosus IgG test and intravenous contrast enhanced computed tomography (CT). He took albendazole (400 mg) 3 times daily; however, the liver lesions continued to grow infiltratively.

His body weight was 80 kg. Standard liver volume based on the West China formula was 1254.6 mL [14]. Preoperative complete blood counts were normal. The liver function was essentially normal. The prothrombin time was 12 s, and the international normalized ratio was 1.3. The indocyanine green (ICG) R15 test was 4.7%. The Child-Pugh score was grade A. His hepatitis B surface antigen was negative.

**Fig. 1:**
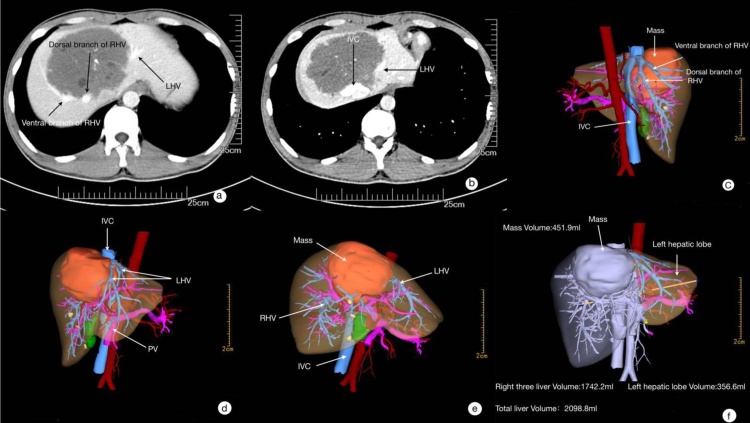
Enhanced abdominal CT and 3-dimensional (3D) imagings analysis. a: the lesion invaded the root of right hepatic vein; b: invaded the root of left hepatic vein; c: invaded the root of right hepatic vein branch (ventral/dorsal branches) and the retrohepatic anterior wall of IVC; d: invaded the root of left hepatic vein (3D image); e: the lesion was adjacent to the porta hepatis (3D image); f: the volume of the left liver section (3D image).

Enhanced abdominal CT showed a large mass, 10 × 9 × 9 cm in liver segments 4, 5, 8. The hepatic veins were invaded (Fig. 1). The right HV, including its ventral/dorsal branches were invaded for about 5 cm. The circumferential diameter of the root was involved for more than 3/4 (Fig. 1a/c). The left HV was invaded for 3 cm, and the circumferential diameter of involvement was 1/4. The retrohepatic anterior wall of inferior vena cava (IVC) was also invaded (Fig. 1b/d). The lesion was adjacent to the porta hepatis. The volume of the whole liver was 2098.8 mL, and the volume of the left lateral liver section (segment 2 and 3), and the segments 4–8 for not including the lesions were 356.6 mL and 1290.3 mL, respectively.

The general condition of the patient was good and there was no contraindications to surgery.

The volume of left lateral liver section (segment 2 and 3) only occupied 17% of the total liver volume and 30% of the standard liver volume (376.4 mL), respectively. The residual liver volume was too small and could lead to postoperative liver failure if segments 4–8 were resected.

For this patient, we decided to carry out in situ split of the liver along the right border of the falciform ligament using the anterior approach, followed by reconstruction of the left portal and left hepatic veins. Then, the remaining liver which included segments 1, 4–8 were excised to carry out ex vivo bench surgery to resect all HAE involved tissues, follow by auxiliary right autologous liver transplantation. The invaded IVC was reconstructed using allogeneic veins with a wide-mouth hepatic vein-IVC anastomosis.

## 3 Operation procedure

**Fig. 2:**
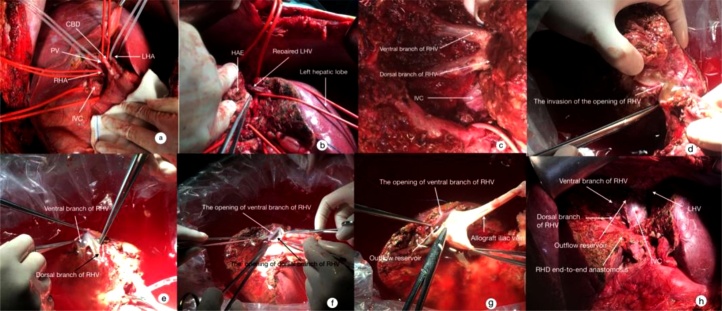
Operative procedure: a: separated the branches of HA/HV/BD, dissected the suprahepatic and subhepatic IVC; b: slit the liver parenchyma based on the anterior approach, and repaired the left hepatic vein during resection; c: dissected down to the invaded roots of right hepatic vein (ventral/dorsal branches) and retrohepatic IVC; d: resected the AE lesion in vitro; e: the remnant right liver after resection of the mass; f: reconstructed the outflow of right liver with a wide mouth anastomosis using allogeneic veins (anterior wall); g: reconstructed the outflow of right liver with a wide mouth anastomosis using allogeneic veins (posterior wall); h: auxiliary right autologous liver transplantation.

The Mercedes Benz incision was used. After mobilization of the liver from the adjacent adhesions, the portal vein (PV) pressure as measured from right gastric epiploic vein intubation was 20.7 mmHg (1 mmHg = 0.133 kPa). The porta hepatis was dissected to isolate the PV, hepatic artery (HA) and common bile duct. The hepatic veins and the IVC were then dissected (Fig. 2a). The lesion was found to invade the roots of the hepatic veins (HV)s. The suprahepatic and subhepatic IVC were dissected. The liver parenchyma was split on the right border of the falciform ligament using the anterior approach with a ultrasonic dissector. The left branch of PV was repaired. The root of the left HV was exposed to show the lesion invasion for about 3 cm length (Fig. 2b). The roots of the right HV (ventral/dorsal branches) were shown to be invaded (Fig. 2c). The middle HV and retrohepatic IVC were also shown to be invaded.

After the patient’s hemodynamic state became stable with occlusion of the IVC and the porta hepatis, the right trisections of the liver, together with segment 1 to include the whole mass and anterior wall of IVC were resected. The specimen was moved into an ice bath for bench surgical resection and outflow reconstruction. The right HA, right PV and right bile duct were divided separately. The explanted liver was perfused with 3000 mL of 4 °C University of Wisconsin (U–W) solution via the right PV branch, and the right HA and right bile duct were simultaneously perfused with 200 mL of U–W solution using syringe injection. The portal pressure in the left lateral section was high. The measured PV pressure was 28 mmHg. However, the patient remained hemodynamically stable. Ex vivo liver resection was performed for the liver AE lesion which had invaded the IVC. Parenchymal liver transection was performed with a minimum of 1.0-cm lesion-free margin using the Cavi-Pulse Ultrasonic Surgical Aspirator (CUSA, Valleylab, Boulder, CO). The autograft was inspected carefully by repeated perfusion to detect any bile leakage or vascular defects. The outflow of the right HV to the IVC was reconstructed using allogeneic veins for a wide mouth anastomosis.

**Fig. 3:**
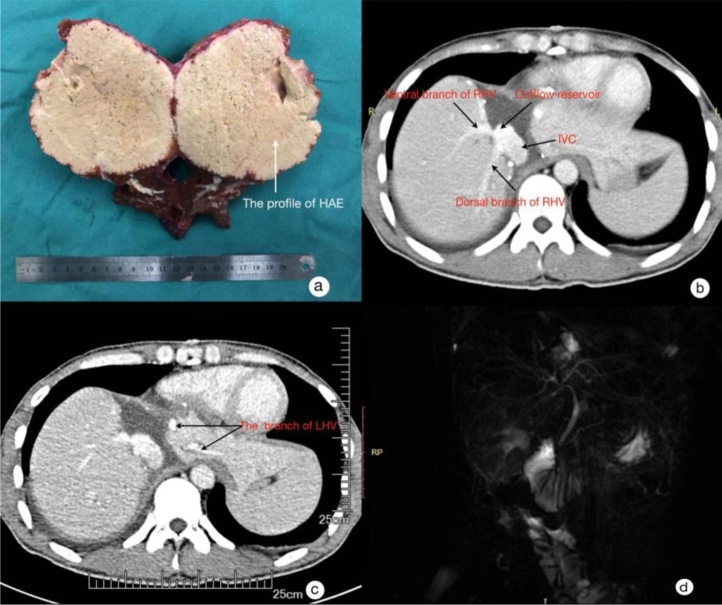
Specimen and images during postoperative follow-up a: the specimen; b: the ventral/dorsal branches and wide mouth anastomosis (1 month after operation); c: the branches of left HV (1 month after operation); d: MRCP (1 month after operation).

The right-PV, right-HA and right-HV were reconstructed using end-to-end anastomosis, respectively. At the end of the operation, the measured PV pressure was 10.9 mmHg. The resected specimen was sent for pathological examination (Fig. 3a).

## 4 Postoperative therapy

**Fig. 4:**
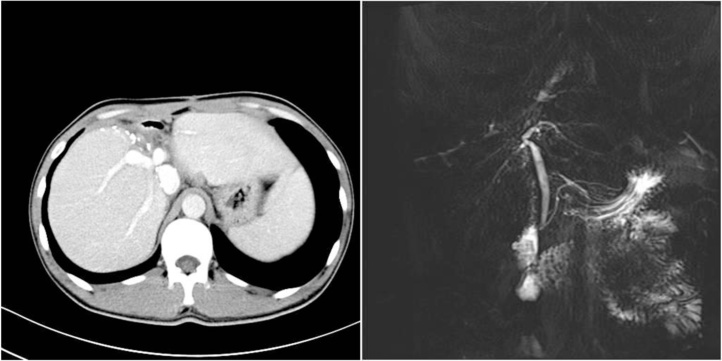
a: CT scan (6 months after operation); b: MRCP (6 months after operation).

The surgical procedure lasted for 12 h, and the blood loss was 800 mL. The anhepatic phase lasted for 1 h. This patient was admitted to the ICU after surgery. A low-molecular-weight heparin sodium injection (2050 IU, 12 h) was started after the patient was stable hemodynamically. The dosage was adjusted according to the weight of the patient and the INR. When the patient began eating, the low-molecular-weight heparin sodium was discontinued and subsequently changed to warfarin sodium tablets (the individual dosage was adjusted according to the weight and the INR of the patient with a reference of 2.0–3.0). This patient was transferred from the ICU to the general ward 3 days after operation. He did not develop any complications. The pathological examination confirmed AE lesion. The CT and MRI 1 month postoperation revealed there was no thrombus in the reconstructed right PV, right HA, right HV and the left HV. After 6 months, CT scan and MRI indicated no hepatic nodules and there was no liver congestion (Fig. 4). There was no stenosis of the reconstructed right bile duct.

## 5 Discussion

Currently, radical resection combined with albendazole therapy is the best treatment for HAE [1,2]. However, intrahepatic vessels and bile ducts could be complexly involved in patients with end-stage HAE, presenting with difficulties for radical resection. Ex-vivo liver resection and autologous liver transplantation (ERAT) using living donor livers have been used for treatment of patients with end-stage hepatic AE [[8], [9], [10], [11]]. When it becomes necessary to reconstruct the IVC or the PV-IVC, an anhepatic phase is required and the injury caused by liver reperfusion, cold storage and ischemia/reperfusion are important factors affecting treatment results [[12], [13], [14], [15]]. For the treatment of end stage HAE in this patient, we initially split the liver using the anterior approach along the right border of the falciform ligament. In special case when the HAE mass is huge and the volume of future liver remnant (FLR) is small, methods including portal vein embolization (PVE) and ALPPS may be used to increase the volume of the FLR, thus creating an opportunity for a second phase liver resection. However, there are limitations for these two methods: ALPPS can cause difficulties in the second phase liver resection by causing adhesions; PVE could not be done in this patient because of AE involvement of right/middle/left hepatic veins, inferior vena cava and left portal vein. Furthermore, for this patient, the S1, S4-S8 segments of the liver were removed for subsequent autologous liver transplantation, whereas the S2 and S3 segments of the left liver were preserved in situ. The subsequent autologous right remnant liver transplantation provided an additional volume of functional liver of about 1200 mL, in addition to the S2 and S3 of the left lateral section. This method of auxiliary autologous liver transplantation for HAE treatment has not been reported before. Furthermore, this modified ERAT procedure reduced the duration of the anhepatic phase compared with the traditional ERAT procedure [15,16].

In this patient, after the right liver was removed, the measured PV pressure rose from 20.7 mmHg to 28 mmHg, and the surface tension of the left liver was high, which indicated the possibility of postoperative small-for-size syndrome [17,18]. After the HAE mass was resected and the right liver remnant was re-implanted, the measured PV pressure became 10.9 mmHg. The surface tension of the right/left liver became normal with adequate bile secretion. This procedure preserved adequate functional liver tissues and reduced the risk of hepatic failure. Also, this procedure limited hepatic ischemia and reperfusion injury because only part of the liver was separated for mass resection, rather than the total liver.

Outflow reconstruction is the most technically difficult part to avoid in ERAT [[19], [20], [21]]. Artificial autologous and allograft vascular grafts, as well as the ligamentum teres have been used for reconstruction. For this case, we used allogeneic veins for hepatic vein reconstruction. The allogeneic vein was obtained 2 weeks ago from a donor with the same blood type and it was stored in 4 °C. We reconstructed the outflow of the right liver with a wide mouth. There was no need for any immunosuppressive therapy. CT and MRI 1 month postoperation revealed there was no thrombus in the reconstructed right PV, right HA, right HV and left HV.

In conclusion, we reported a new surgical procedure to treat a patient with advanced HAE. The procedure helped to maintain the stability of the systemic and portal circulation without the use of a veno-venous bypass. It provided part of liver function during the operation, preserved adequate functional liver parenchyma and reduced the risk of hepatic failure.

## Declaration of Competing Interest

No any conflicts of interest.

## Sources of funding

This work was supported by grants from Chendu Branch, Chinese Academy of Sciences, Health Department of Sichuan Province, China (No. 150192) and Funding of Sichuan Academy of Medical Sciences.

## Ethical approval

This case was approved by the ethical committee of the Sichuan Academy of Medical Sciences (Sichuan Provincial People’s Hospital), and it followed the ethical guidelines of the 1975 Declaration of Helsinki.

## Consent

Written informed consent was obtained from the patient for publication of this case report.

## Author contribution

Wan Yee Lau and Shaoping Deng have contributed equally to this work; Wan Yee Lau, Shaoping Deng and Yu Zhang designed the study; Chong Yang, Hongji Yang, Jun Liu, Guo Zhou and Di Xian collected the patient’s clinical data; Yu Zhang, Eric C.H. Lai and Wan Yee Lau analyzed the data and wrote the paper.

## Registration of research studies

Researchregistry5206.

## Guarantor

Yu Zhang, Wan Yee Lau.

## Provenance and peer review

Editorially reviewed, not externally peer-reviewed.
